# Annotation of Secretariat and Hydrus, two DJ cluster phages isolated on *Gordonia rubripertincta*

**DOI:** 10.1128/MRA.00623-23

**Published:** 2023-09-26

**Authors:** Jackson Bland, Sydney Miller, Elijah D. Algarin-Martinez, Abbigail M. Biggs, Maria E. D. Cavasini, Michael A. Chase, Caitlyn Coleman, Victoria Correa, Don F. Danielson, Wynter R. Dean, Joseph L. French, Mae E. Horne, Breanna M. Macumber, Francesca Kristal Martini, Sydney G. Mazzei, Coen E. E. McGarrah, Oslow Odegaard, Indu S. Parameswaran, Corrisa Quarterman, Taylor M. Rand, Kira M. Ruiz-Houston, Ava R. Sciacchitano, Nina S. Seidensticker, Anna Soltys, Adrian E. Terron-Osorio, Allyson L. Todd, Audrey R. Wood, Maxwell D. Ungrey, Richard S. Pollenz

**Affiliations:** 1 Department of Molecular Biosciences, University of South Florida, Tampa, Florida, USA; California State University San Marcos, San Marcos, California, USA

**Keywords:** actinobacteria, *Gordonia rubripertincta*, bacteriophage genetics, host range

## Abstract

Secretariat and Hydrus are phages grouped into the DJ cluster that were isolated on *Gordonia rubripertincta* NRRL B-16540. The phages have 75% nucleotide identity and share 73% gene content. Secretariat has a genome with 84 predicted genes, while Hydrus has 91 predicted genes and can also infect *Gordonia terrae* 3612.

## ANNOUNCEMENT

There are an estimated 10^31^ phage particles in the world (1), and the SEA PHAGES program has isolated and annotated nearly 4,500 actinobacteriophage genomes (2, 3). The isolation of evolutionarily diverse actinobacteriophages can help advance the understanding of phage genomics and is an important step in providing phages that may be used in environmental and health applications.

Secretariat and Hydrus were isolated from moist soil samples taken from Lutz and Tampa, FL, respectively. GPS coordinates are shown in Table 1. Soil samples were mixed with equal parts peptone-yeast calcium media (PYCa), shaken at 250 rpm for 2 h at 25°C, and sterile filtered (0.2 µm PES). *Gordonia rubripertincta* NRRL B-16540 was infected with sterile soil lysates or pure phage and plated on PYCa agar at 30°C. Genomic DNA was isolated after three rounds of plaque purification using the Wizard DNA clean-up kit (A7280; Promega). Genomic DNA was used to create sequencing libraries with the NEB Ultra II Library Kit, v3 Reagents. Sequencing was performed by the Pittsburgh Bacteriophage Institute, and the library ran on an Illumina MiSeq instrument. The SRA summary data for each phage is presented in Table 1. Raw reads were assembled with Newbler (v2.9) (4), yielding a single contig for both phages. The results were checked for completeness, accuracy, and genome termini using Consed (v29.0) (5). Default parameters were used for all software unless otherwise specified. Both Secretariat and Hydrus have 3′ sticky overhangs (CGCCGCTCT) and were bioinformatically linearized such that base 1 is assigned in accordance with other *Gordonia* phages (6). Both phages were auto-annotated using DNA master (v5.23.6) (7), and the genes were then manually validated for correct starts and functional calls. GeneMark (v2.5) (8) and Glimmer (v3.02) (9) were utilized to assess start sites and coding potential, and Starterator (v1.2) (3) to summarize the starts across each family of phage genes. Evidence to support a gene product function was collected using HHpred (databases: PDB_mmCIF70_18_jun; Pfam-A_v35; NCBI_Conservd_Domains(CD)_v3.19) (10, 11), and NCBI BLAST (BLAST +2.13) (12). Putative transmembrane domains (TM) were identified using Deep TMHMM (v1.0.24) (13) and TOPCONS (v2.0) (14). Information on each phage regarding isolation, characterization, and gene content is archived in Phamerator (15) and the Actinobacteriophage Database at PhagesDB.org (2; https://phagesdb.org/phages/Hydrus/ and https://phagesdb.org/phages/Secretariat/).

**TABLE 1:** Secretariat and Hydrus SRA and genome data

	SRA data						Genome data				
	Design	Pair end read size	Pairedend reads	Average coverage	Location found	Sampling date	Genome size (bp)	GC content (%)	Total ORFs	Average nucleotide identity to Hydrus	Percent gene content to Hydrus
Secretariat	NEB Ultra II Library Kit, v3	150 bp	350,906	854	Lutz, FL, 27.15559 N, 82.43884 W	9/5/2019	57,371	52.8	84	75.27%	73.26%
Hydrus	NEB Ultra II Library Kit, v3	150 bp	276,062	510	Tampa, FL, 28.09200 N, 82.38900 W	9/8/2020	60,842	51.8	91		

**Fig 1:**
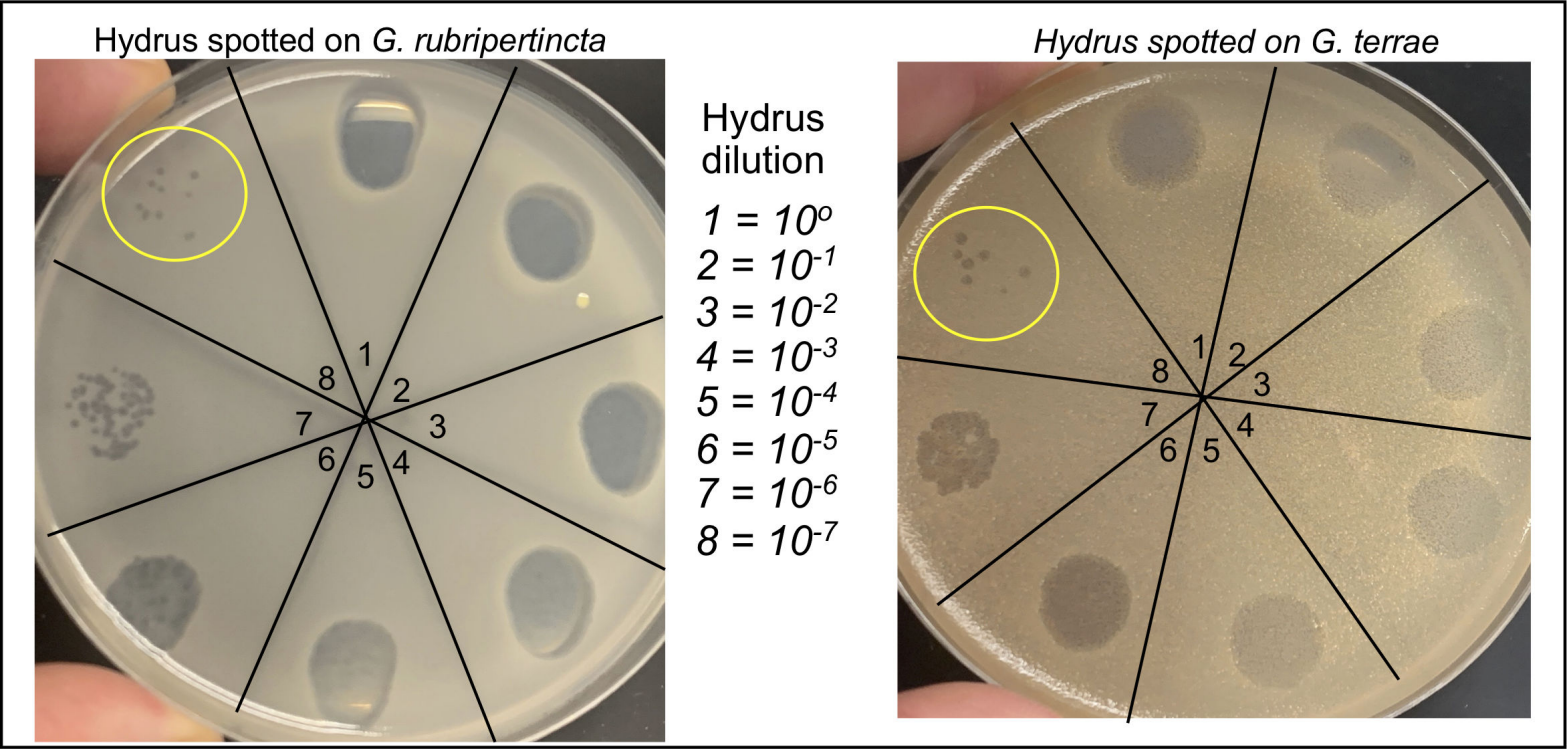
Spot titer plates of Hydrus on a lawn of *G. rubripertincta* and *G. terrae*. Bacteria were mixed with PYCa top agar supplemented with 1 mM CaCl_2_ and plated onto PYCa agar. A Hydrus lysate of 1 × 10^10^ PFU/mL was serially diluted to 10^−7^ and 10 uL of each dilution spotted onto the hardened top agar. Plates were incubated at 30°C for 36 h. Titers were calculated by counting individual plaques (yellow circles). In this representative experiment, the Hydrus titer from the *G. rubriupertincta* lawn was 9.0 × 10^9^ PFU/mL and 7.0 × 10^9^ PFU/mL on the *G. terrea* lawn. The efficiency of plating was 7.0 × 10^9^ PFU/mL/9.0 × 10^9^ PFU/mL = 0.78.

Secretariat and Hydrus are siphoviruses with 60 nm icosahedral capsids and flexible tails of ~215 nm, as determined by electron microscopy. The Secretariat genome is 57,371 bp and contains 84 predicted protein-coding genes. Hydrus is 60,842 bp with 91 predicted protein-coding genes due to the presence of several regions of nucleotide insertion that add additional genes to the genome. Overall, the two phages share 62 common genes, with 23 having a known function. Hydrus has 29 unique genes compared to the Secretariat, of which 95% encode proteins with no known function. Each phage is grouped into the DJ cluster with 33 other phages that infect either *G. rubripertincta or Gordonia terrae* strains*.* Host range analysis shows that Hydrus, but not Secretariat, can infect the *G. terrae* 3612 strain with an efficiency of plating of ~1.0 (Fig. 1).

## Data Availability

The Secretariat whole Genome Shotgun project has been deposited in DDB/ENA/GenBank under accession no. MT310850 and SRX20165781. Hydrus whole Genome Shotgun project has been deposited in DDB/ENA/GenBank under the accession no. OQ938589 and SRX19690838. The versions described in this paper are the first.
